# Hidradenitis suppurativa and familial Mediterranean fever: a report of 6 cases and literature review

**DOI:** 10.1186/1546-0096-13-S1-P105

**Published:** 2015-09-28

**Authors:** S Abbara, S Georgin-Lavialle, G Grateau, C Bachmeyer, D Buob, P Senet, S Audia, V Delcey, O Steichen, J-P Bastard, S Fellahi, S Amselem, K Stankovic Stojanovic

**Affiliations:** 1 AP-HP, Internal Medicin, Paris, France

## Introduction

Familial Mediterranean fever (FMF) is the most frequent monogenic autoinflammatory disease (AID). Hidradenitis suppurativa (HS) is an inflammatory skin disease characterized by recurrent abscesses and scarring in apocrine gland-bearing areas. HS shares common features with AID including a positive correlation between the increase of acute phase reactants (APR) and the severity of the disease. HS can be associated with other AID.

## Objectives

To describe a series of patients who had both HS and FMF.

## Patients and methods

Descriptive observational retrospective study relating the main characteristics of patients who had HS among the FMF cohort followed in the French adult reference center.

## Results

Among 151 FMF adult patients, we identified 6 patients with HS, 2 women, with a median age of 36 years [range 27-70]. Median age for first symptoms of FMF was 11.5 years [3-30], and for colchicine administration 20.5 years [4-34] with a mean dosage at the time of the study of 1.25mg/day [1-2]. HS was diagnosed at a median age of 31.5 years [13-63]. Treatment consisted in consecutive antibiotics (5 patients), surgery (3 patients), and no treatment in one recent diagnosed patient. There was no concurrence between HS and FMF flares. APR were normal in 3 patients. For the 3 remaining patients: one took irregularly 1mg/d of colchicine and had no FMF flares but an active HS; the second one had 1.5mg/d of colchicine and the third patient 2mg/d, both had active FMF and HS. For the older patient of the series, FMF occurred at the age of 10 years and HS at 15 years; AA amyloidosis was diagnosed when he was 69 years old because of proteinuria and increased APR whereas he took 1mg/d of colchicine, thus he received interleukin (IL)-1 inhibitor in association with colchicine.

In the literature, IL-1 inhibitor demonstrated an efficacy in 9 out of 12 patients with HS and no AID. Three cases of AA amyloidosis associated with HS were reported.

## Conclusion

FMF and HS are suspected to share common pathways to the activation of inflammasome and the secretion of IL-1 beta. The association of FMF and HS could induce a greater and protracted secretion of IL-1 beta and enhance the risk of earlier occurrence of AA amyloidosis. Thus, APR should be carefully monitored in patients with FMF and HS and treatment should be intensified if needed in order to prevent AA amyloidosis.

